# The impact of SGLT2 inhibitors on αKlotho in renal MDCK and HK-2 cells

**DOI:** 10.3389/fendo.2023.1069715

**Published:** 2023-03-08

**Authors:** Lisa Wolf, Michael Föller, Martina Feger

**Affiliations:** University of Hohenheim, Department of Physiology, Stuttgart, Germany

**Keywords:** SGLT2 inhibitors, αKlotho, nephroprotection, cardioprotection, calcitriol (1,25(OH)_2_D_3_)

## Abstract

αKlotho is a transmembrane protein predominantly expressed in the kidney serving as a co-receptor for phosphate homeostasis-regulating hormone FGF23 and has an extracellular domain that can be cleaved off and is a hormone. αKlotho deficiency results in accelerated aging and early onset of aging-associated diseases while its overexpression strongly expands the lifespan of mice. Moreover, αKlotho exerts health-beneficial anti-inflammatory, anti-neoplastic, anti-fibrotic, and anti-oxidant effects. Higher αKlotho levels are associated with better outcomes in renal and cardiovascular diseases. SGLT2 inhibitors are novel drugs in the treatment of diabetes by inhibiting renal glucose transport and have additional nephro- and cardioprotective effects. We explored whether SGLT2 inhibitors affect αKlotho gene expression and protein secretion. Experiments were performed in renal MDCK and HK-2 cells, and αKlotho transcripts were determined by qRT-PCR and Klotho protein by ELISA. SGLT2 inhibitors canagliflozin, sotagliflozin, and dapagliflozin enhanced whereas empagliflozin reduced αKlotho gene expression in MDCK cells. By the same token, canagliflozin, sotagliflozin, dapagliflozin, but not empagliflozin down-regulated p65 subunit of pro-inflammatory NFκB. In HK-2 cells, all SGLT2 inhibitors reduced αKlotho transcripts. Canagliflozin and sotagliflozin, however, increased Klotho protein concentration in the cell culture supernatant, an effect paralleled by up-regulation of ADAM17. Taken together, our investigations demonstrate complex effects of different SGLT2 inhibitors on αKlotho gene expression and protein secretion in renal MDCK and HK-2 cells.

## Introduction

αKlotho is a renal protein the lack of which results in a phenotype recapitulating human aging: αKlotho-deficient mice die at an age of a few weeks only whilst exhibiting aging-associated diseases affecting almost all organs including emphysema, hearing loss, infertility, or hypogonadism amongst others (1–3). The animals exhibit massive hyperphosphatemia and excess of active vitamin D, 1,25(OH)_2_D_3_, with a low-phosphate diet or low-vitamin D diet normalizing their phenotype (4, 5). Hence, further research has demonstrated a pivotal role of αKlotho in maintaining phosphate homeostasis: αKlotho is a transmembrane protein that enhances binding affinity of fibroblast growth factor 23 (FGF23) for its renal receptor (6, 7). FGF23 is produced in bone as a hormone and reduces production of 1,25(OH)_2_D_3_ and reabsorption of phosphate, thereby lowering serum levels of 1,25(OH)_2_D_3_ and phosphate (8–11). The joint action of FGF23 and αKlotho in the regulation of phosphate and 1,25(OH)_2_D_3_ explains why FGF23 deficiency is comparable to αKlotho deficiency in mice (12–14). In addition to being the co-receptor of FGF23, a soluble form of αKlotho exists called sKL which results from the cleavage of transmembrane αKlotho by proteases ADAM10 and ADAM17 (15, 16). SKL has hormone-like properties and influences various cellular effects including signaling or membrane transport (17).

In general, the effects of αKlotho are beneficial: Also by inhibiting insulin-like-growth factor 1 (IGF-1) or Wnt signaling, αKlotho exerts anti-tumor effects (18, 19). αKlotho has anti-inflammatory and anti-oxidant properties (20–22). Notably, αKlotho is organoprotective: It protects from stress-induced cardiac hypertrophy through suppression of transient receptor potential cation channel subfamily C member 6 (TRPC6) (23) or from ischemia/reperfusion injury (24). Moreover, αKlotho is nephroprotective and has proven beneficial in diabetic nephropathy (25, 26). It suppresses kidney fibrosis and delays progression of chronic kidney disease (CKD) (27–31). All these beneficial effects are likely to contribute to the 30% longer lifespan in mice overexpressing αKlotho (2).

SGLT2 inhibitors such as dapagliflozin, canagliflozin, empagliflozin, or sotagliflozin were introduced as new antidiabetics in the early 2010s (32). They efficiently lower blood glucose levels by inhibiting renal SGLT2, a Na^+^-dependent glucose transporter which normally accomplishes complete glucose reabsorption along with SGLT1, another isoform that is not inhibited by specific SGLT2 inhibitors (33–35). As a consequence, the patients excrete more glucose in their urine (36, 37). Recent large clinical studies of high quality have unequivocally proven that SGLT2 inhibition has further significant and diabetes-independent cardio- and nephroprotective effects that are, in many aspects, regarded as a milestone in cardiology and nephrology (38–40). SGLT2 inhibition is the only approved drug therapy in heart failure with preserved ejection fraction (HFpEF), and in CKD, SGLT2 inhibitor therapy is the first major breakthrough since the introduction of angiotensin-converting enzyme (ACE) inhibitors in the 1990s, since the drugs convincingly delay loss of kidney function (41–43). The precise underlying mechanisms of SGLT2 inhibitor-dependent cardio- and nephroprotection have, hitherto, remained enigmatic although many beneficial effects have been characterized including the prevention of hyperfiltration in the kidney or anti-inflammatory and anti-fibrotic effects (44, 45).

Given the strong cardio- and nephroprotection provided by αKlotho, which is also, at least in part, dependent on its anti-inflammatory and anti-fibrotic effects, we hypothesized that benefits of therapy with SGLT2 inhibitors may be related to up-regulation of cellular αKlotho levels. This hypothesis has already been formulated in a recent review (46). To verify it, we performed experiments in canine MDCK and human HK-2 kidney cell lines.

## Methods

### Cell culture and treatments

Madin Darby Canine Kidney Cells (MDCK) (NBL-2) (CVCL_0422, CLS Cell Lines Service, Eppelheim, Germany) were grown in Dulbecco’s Modified Eagle Medium: Nutrient Mixture F-12 (DMEM/F-12; Gibco, Life Technologies, Darmstadt, Germany) supplemented with 5% fetal bovine serum (FBS), 2 mM glutamine, 100 U/mL penicillin, and 100 μg/mL streptomycin (all from Gibco). Human kidney 2 (HK-2) cells (CRL-2190, ATCC, Manassas, VA, USA) and rat osteoblast-like UMR-106 cells (CRL-1661, ATCC) were cultured in DMEM of high glucose (Gibco) containing 10% FBS, 100 U/mL penicillin, and 100 μg/mL streptomycin. Cells were incubated at 37°C and 5% CO_2_.

For experiments, cells (150,000 MDCK or 120,000 HK-2 cells per well) were seeded into 6-well plates, grown for 24 h, and then treated for 24 h with or without canagliflozin, sotagliflozin, empagliflozin (all from Selleck Chemicals, Planegg, Germany), dapagliflozin, or phlorizin dihydrate (both from Sigma-Aldrich, Schnelldorf, Germany) at the indicated concentrations. Control cells were treated with the appropriate amount of solvent dimethyl sulfoxide. For the measurement of soluble Klotho protein in the cell culture medium, supernatants were collected after 24 h of treatment and stored at -70°C until further use.

UMR-106 cells were plated at a density of 200,000 cells per well in growth medium supplemented with 10 nM 1,25(OH)_2_D_3_ (Tocris, Bio-Techne, Wiesbaden-Nordenstadt, Germany). After 24 h, cells were treated with SGLT2 inhibitor canagliflozin, sotagliflozin, empagliflozin, dapagliflozin, or vehicle only for 24 h.

### RNA isolation and qualitative expression analysis

Total RNA from MDCK, HK-2, and UMR-106 cells was isolated using a phenol-chloroform extraction technique (RNA-Solv Reagent, Omega Bio-Tek, Norcross, GA, USA or TriFast Reagent, VWR, Bruchsal, Germany) according to the manufacturer’s protocol. For qualitative detection of *SLC5A1* and *SLC5A2* transcripts and quantitative gene expression analysis in MDCK cells, total RNA was extracted by means of phenol-chloroform-extraction, subjected to DNase treatment, and RNA purification (NucleoSpin RNA, Macherey-Nagel, Düren, Germany). Synthesis of complementary DNA (cDNA) was performed using 1.2 µg of total RNA, random primers, and GoScript Reverse Transcription System (Promega, Mannheim, Germany).

Polymerase chain reaction (PCR) was conducted on a Biometra TAdvanced thermocycler (Analytik Jena, Jena, Germany) using 2 µL cDNA, 0.25 µM canine forward and reverse primers or 0.5 µM human primers, respectively, 10 µL GoTaq Green Master Mix (Promega), and sterile water up to 20 µL. Conditions for PCR included 3 min at 94°C; followed by 40 cycles of 94°C for 30 s; 57°C (for human *SLC5A2*), 59°C (for canine *SLC5A1*) or 60°C (for canine *SLC5A2* and human *SLC5A1*) for 30 s; and 72°C for 30 s.

The following primers (5’→3’ orientation) were used for qualitative and quantitative PCR analysis of SGLT1/2:

*SLC5A1* (canine):

GTGCAGTCAGCACAAAGTGG,

CGGGACACCCCAATCAGAAA;

*SLC5A2* (canine):

CTCTTTGCCAGCAACATCGG,

CACGAACAGCGCATTCCAC;

*SLC5A1* (human):

GCTGCCACCATGGACAGTAG,

AGATGGGGACAAACAGCCAG;

*SLC5A2* (human):

GGAGATGAATGAGCCCCAGG,

TCATGAGCAGGGCATTGAGG.

Amplified PCR products, no reverse transcriptase controls (NRT), and no template controls (NTC) were separated (complete PCR reaction mixture of MDCK samples and 12 µL of the HK-2 samples, respectively), on a 1.5% agarose gel and visualized by ethidium bromide staining.

### Quantitative real-time polymerase chain reaction

Quantitative real-time PCR (qRT-PCR) was performed on a CFX Connect Real-Time PCR System (Bio-Rad Laboratories, Feldkirchen, Germany). The qRT-PCR reaction mixture consisted of 10 µL GoTaq qPCR Master Mix (Promega), 2 µL of cDNA, specific primers and sterile water up to 20 µL. The qRT-PCR conditions were as follows: 95°C for 2 min; 40 cycles of 95°C for 10 s; 56°C for 30 s (canine *KL*), 57°C for 30 s (canine *RELA*), 58°C for 30 s (rat *Fgf23* and *TATA-box binding protein* (*Tbp*)), 59°C for 30 s (human *KL*, *ADAM10*, and *TBP*), 60°C for 30 s (canine *TBP* and human *ADAM17*); and 72°C for 25 s.

The following primers (5’→3’ orientation) were used for qRT-PCR analysis:

*KL* (canine):

AAATGAAGCTCTGAAAGCC,

AATGATAGAGGCCAAACTTC;

*TBP* (canine):

CCTATTACCCCTGCCACACC,

GCTCCCGTACACACCATCTT;

*RELA* (canine):

AACAGCGTGGGGACTATGAC,

GGGCACGGTTGTCAAAGATG;

*ADAM10* (human):

GACCACAGACTTCTCCGGAAT,

TGAAGGTGCTCCAACCCAAG;

*ADAM17* (human):

GGGCAGAGGGGAAGAGAGTA,

TGTGGAGACTTGAGAATGCGA;

*KL* (human):

TGGAAACCTTAAAAGCCATCAAGC,

CCACGCCTGATGCTGTAACC;

*TBP* (human):

TGCACAGGAGCCAAGAGTGAA,

CACATCACAGCTCCCCACCA,

*Fgf23* (rat):

TAGAGCCTATTCAGACACTTC,

CATCAGGGCACTGTAGATAG;

*Tbp* (rat):

ACTCCTGCCACACCAGCC,

GGTCAAGTTTACAGCCAAGATTCA.

Relative mRNA transcript levels were calculated by the 2^-ΔΔCt^ method using *TBP* as internal reference gene and normalized to the control group. For quantitative analysis of SGLT1 and SGLT2 expression, relative mRNA transcripts were calculated using the 2^-ΔCt^ formula and *TBP* as internal reference.

### Klotho protein determination in cell culture supernatant

HK-2 supernatants were subjected to enzyme-linked immunosorbent assay (ELISA) for measurement of soluble Klotho protein (IBL, Hamburg, Germany).

### Statistics

Data are reported as means ± standard error of mean (SEM) in bar graphs with scatter plots, and *n* is the number of independent experiments. Data were tested for normality using Shapiro-Wilk test. Differences between control and treatment group were assessed by two-tailed paired Student’s *t* test, Wilcoxon matched-pairs signed rank test (for data not passing normality), one-sample *t* test or Wilcoxon signed rank test (for data not passing normality), as indicated in the figure legends. *P* < 0.05 was considered statistically significant. SPSS version 27.0 (IBM, Armonk, NY, USA) or GraphPad Prism 6.0 (GraphPad Software, San Diego, California, USA) were used for statistical analysis.

## Results

**Figure 1:**
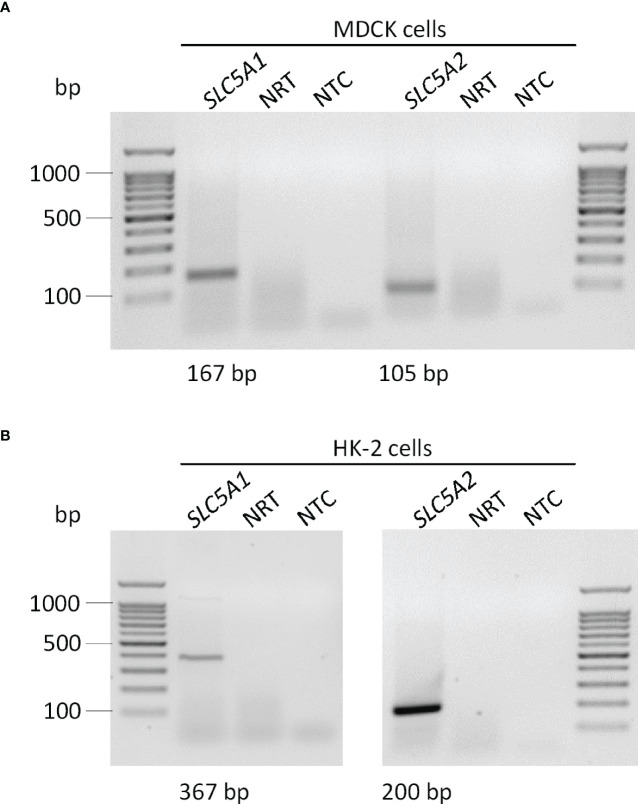
Qualitative analysis of SGLT1 (*SLC5A1*) and SGLT2 (*SLC5A2*) mRNA abundance in MDCK and HK-2 cells. Original agarose gel photo showing *SLC5A1*- and *SLC5A*2-specific PCR products in untreated MDCK **(A)** and HK-2 **(B)** cells. base pair, bp; no template control, NTC; no reverse transcriptase, NRT.

We utilized canine MDCK and human HK-2 kidney cells to study the regulation of αKlotho. At first, we investigated the expression of SGLT1 (encoded by *SLC5A1*) and SGLT2 (encoded by *SLC5A2*) in the two cell lines by PCR. According to qualitative analysis, mRNA specific for both glucose transporters was detectable in MDCK cells seemingly to a small extent (**Figure 1A**). For HK-2 cells, mRNA for both transporters could be verified with a markedly stronger band for SGLT2 (**Figure 1B**). In addition, we performed quantitative analysis and examined relative expression (n=3 for all analyses) of SGLT1 and SGLT2 in both cell lines by qRT-PCR. As a result, cycle threshold (Ct) value for canine *SLC5A1* (SGLT1) in MDCK cells was 37.46 ± 1.499, whereas a positive signal for *SLC5A2* (SGLT2) could not be detected in these cells (Ct > 40 for *SLC5A2*). In HK-2 cells, Ct value for *SLC5A1* (SGLT1) was 37.39 ± 0.661 resulting in 3.93 x 10^-5^ ± 2.055 x 10^-5^ arbitrary units of relative *SLC5A1* mRNA transcription normalized to the housekeeping gene *TBP*. For *SLC5A2* (SGLT2), Ct value was 30.35 ± 0.128 resulting in 4.04 x 10^-3^ ± 0.447 x 10^-3^ arbitrary units of relative *SLC5A2* mRNA transcription normalized to *TBP*.

**Figure 2:**
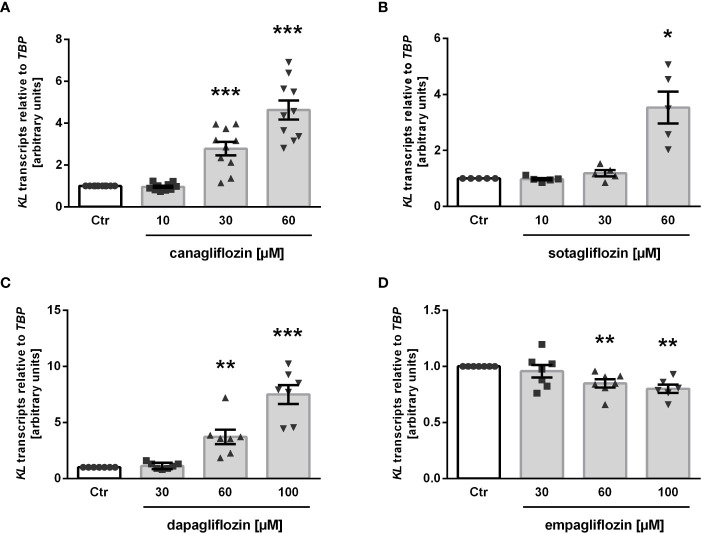
SGLT2 inhibition affects *KL* mRNA expression in MDCK cells. Arithmetic means ± SEM of relative *KL* mRNA abundance normalized to *TBP* in MDCK cells treated without (Ctr) or with canagliflozin (**A**; n=7), sotagliflozin (**B**; n=5), dapagliflozin (**C**; n=7), or empagliflozin (**D**; n=6-7) for 24 h. *p < 0.05, **p < 0.01 and ***p < 0.001 indicate significant difference from control cells. (One sample *t* test).

Next, we treated MDCK cells with increasing concentrations of the specific SGLT2 inhibitor canagliflozin, which is widely used in the treatment of patients, for 24 h and utilized qRT-PCR to assess the effect on αKlotho gene expression. As detailed in **Figure 2A**, canagliflozin up-regulated αKlotho gene expression in a dose-dependent manner. As a further step, we investigated whether also sotagliflozin, a dual SGLT1/SGLT2 inhibitor approved for use in patients, up-regulates αKlotho. To this end, we treated MDCK cells with different concentrations of sotagliflozin for 24 h, and measured αKlotho transcripts by qRT-PCR. Similar to canagliflozin, sotagliflozin enhanced αKlotho mRNA abundance (**Figure 2B**). Further experiments explored the effect of a 24 h-incubation with or without dapagliflozin (**Figure 2C**) or empagliflozin (**Figure 2D**) on αKlotho in MDCK cells. Whereas dapagliflozin (**Figure 2C**) up-regulated αKlotho gene expression to an extent similar to canagliflozin (**Figure 2A**) or sotagliflozin (**Figure 2B**), empagliflozin surprisingly reduced αKlotho transcripts in MDCK cells (**Figure 2D**).

**Figure 3:**
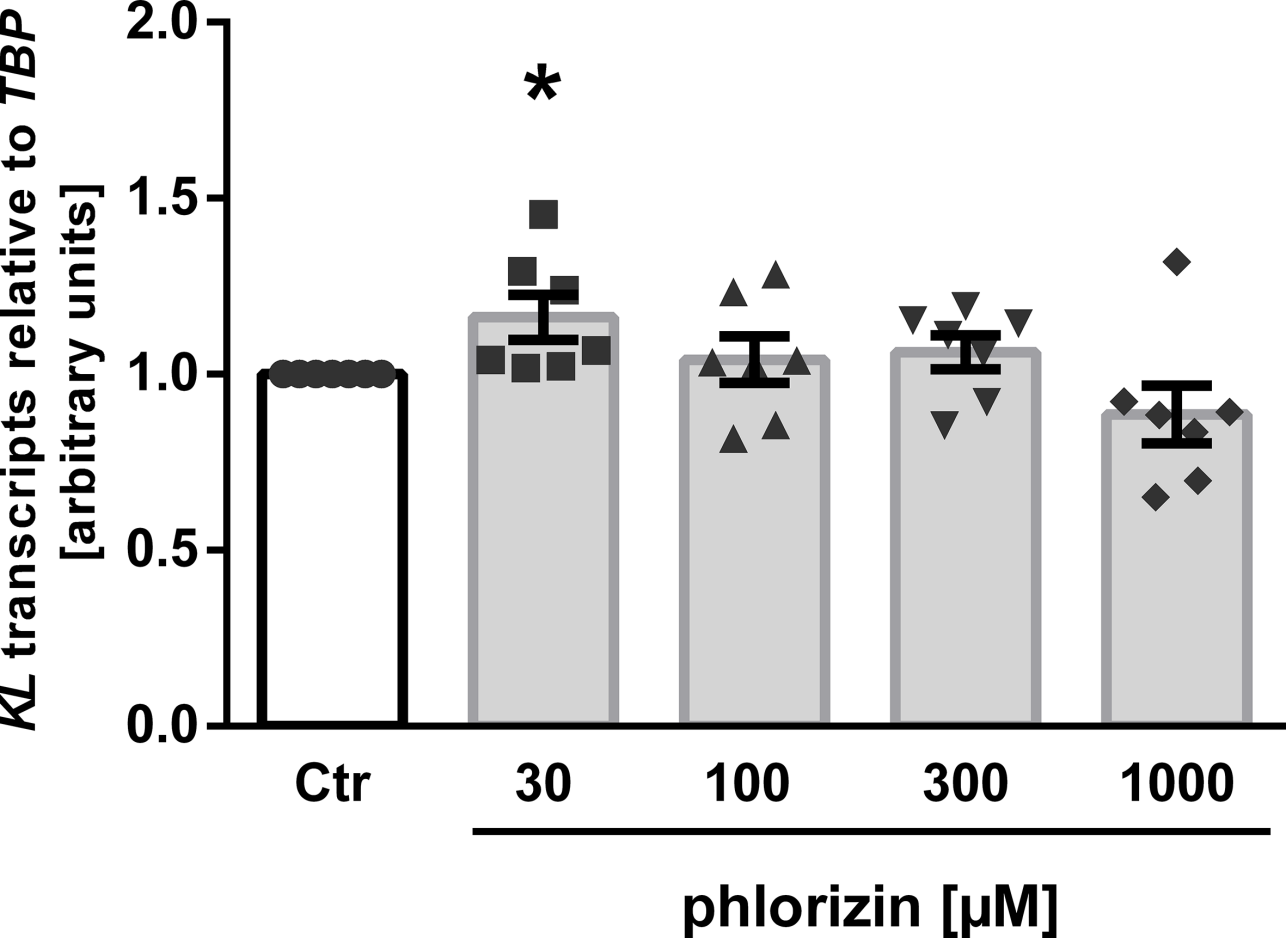
Effect of phlorizin on *KL* transcript level in MDCK cells. Arithmetic means ± SEM of relative *KL* mRNA abundance normalized to *TBP* (n=7) in MDCK cells treated without (Ctr) or with phlorizin for 24 h. *p < 0.05 indicates significant difference from control-treated cells. (One sample *t* test).

Finally, we tested phlorizin, a non-specific glucose transporter inhibitor inhibiting both, SGLT1 and SGLT2, which is not approved for use in patients. As illustrated in **Figure 3**, treatment of MDCK cells with 30 µM phlorizin (24 h) resulted in a small, but statistically significant up-regulation of αKlotho transcripts whereas higher concentrations had no significant effect on αKlotho (**Figure 3**).

**Figure 4:**
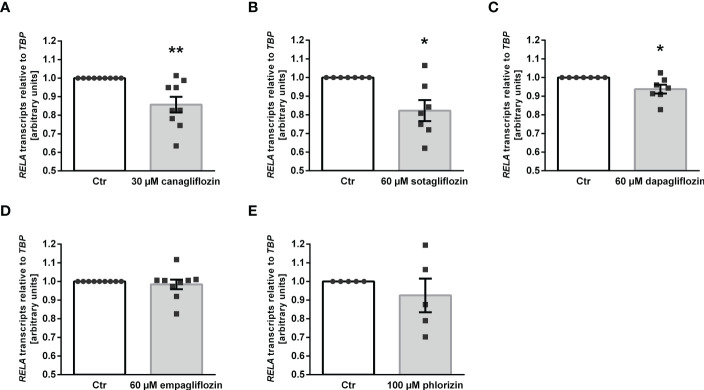
Reduced expression of NFκB subunit p65 (*RELA*) following SGLT2 inhibitor treatment in MDCK cells. Arithmetic means ± SEM of relative *RELA* mRNA expression normalized to *TBP* in MDCK cells treated with vehicle (Ctr), canagliflozin (**A**; n=9), sotagliflozin (**B**; n=7), dapagliflozin (**C**; n=7), empagliflozin (**D**; n=9) or with phlorizin (**E**; n=5) for 24 h. *p < 0.05 and **p < 0.01 indicate significant difference from control-treated cells. (One sample *t* test).

Owing to its anti-inflammatory properties (45), we investigated whether SGLT2 inhibitors impact on the expression of p65 subunit (encoded by *RELA*) of pro-inflammatory transcription factor complex NFκB. As illustrated in **Figure 4**, the three SGLT2 inhibitors which enhanced αKlotho down-regulated *RELA* expression in MDCK cells (**Figures 4A–C**). Empagliflozin, which did not significantly affect αKlotho, and phlorizin did not significantly modify *RELA* in MDCK cells (**Figures 4D, E**).

**Figure 5:**
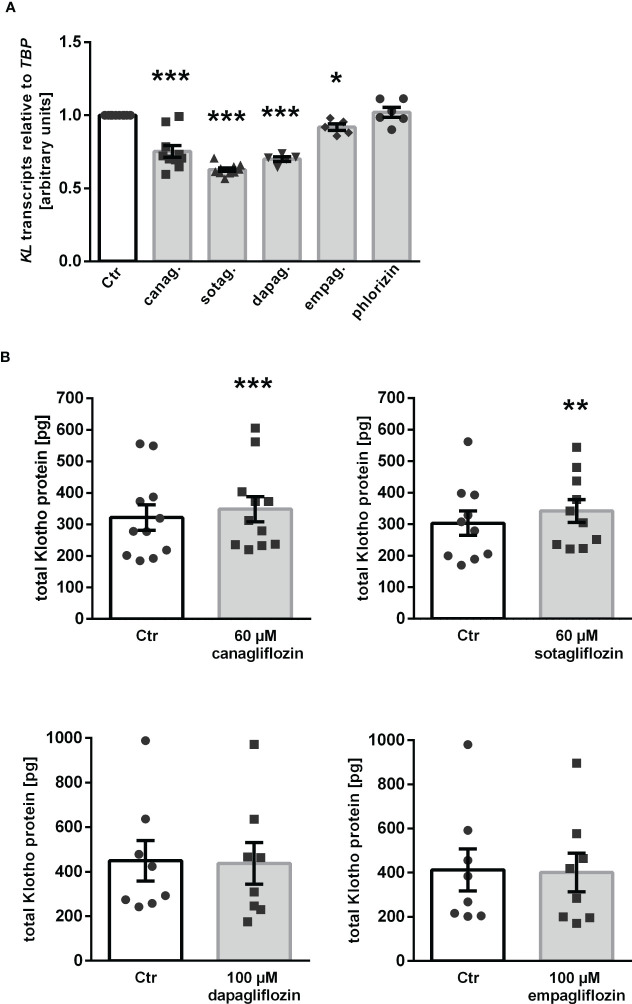
Impact of SGLT2 inhibition on Klotho production in HK-2 cells. **(A)** Arithmetic means ± SEM of relative *KL* mRNA expression in HK-2 cells normalized to *TBP* treated either with vehicle (Ctr), canagliflozin (60 µM; n=10), sotagliflozin (60 µM; n=10), dapagliflozin (100 µM; n=5), empagliflozin (100 µM; n=5), or phlorizin (100 µM; n=6) for 24 h. **(B)** Arithmetic means ± SEM of Klotho protein (pg) in the supernatant of HK-2 cells either treated with vehicle (Ctr), canagliflozin (n=11), sotagliflozin (n=10), dapagliflozin (n=8), or empagliflozin (n=8) for 24 h. *p < 0.05, **p < 0.01, and ***p < 0.001 indicate significant difference from control-treated cells. (A: One sample *t* test; B: Wilcoxon matched-pairs signed rank test (upper panel, right) or two-tailed paired Student’s *t* test).

Our results thus far indicate that canagliflozin, sotagliflozin, and dapagliflozin, but not empagliflozin, up-regulated αKlotho in MDCK cells. We wondered whether this effect translates into enhanced Klotho protein secretion. In order to answer this question, we carried out a new series of experiments in human HK-2 cells since ELISA-based Klotho protein quantification is not feasible in canine MDCK cells. First, we examined the effect of the SGLT2 inhibitors on αKlotho transcripts in HK-2 cells. As demonstrated in **Figure 5A**, canagliflozin, sotagliflozin, dapagliflozin as well as empagliflozin down-regulated αKlotho gene expression in HK-2 cells in sharp contrast to MDCK cells, whereas phlorizin had no significant effect on αKlotho mRNA levels in HK-2 cells. As a second step, we employed ELISA to measure Klotho protein in the cell culture supernatant. According to **Figure 5B**, Klotho protein abundance was slightly but significantly higher in the supernatant of HK-2 cells treated with canagliflozin or sotagliflozin compared to control cells (**Figure 5B**, upper panel). Dapagliflozin or empagliflozin did, however, not significantly modify the Klotho protein amount in the cell culture supernatant (**Figure 5B**, lower panel).

**Figure 6:**
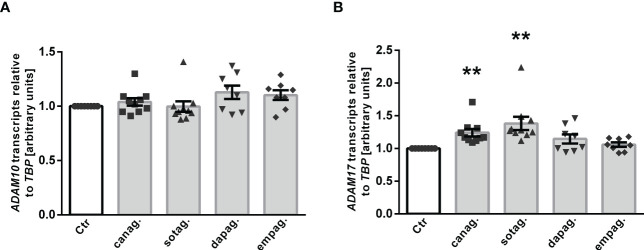
Effect of SGLT2 inhibition on *ADAM10* and *ADAM17* mRNA expression in HK-2 cells. Arithmetic means ± SEM of relative *ADAM10* **(A)** and *ADAM17* **(B)** mRNA expression normalized to *TBP* in HK-2 cells either treated with vehicle control (Ctr), canagliflozin (60 µM; n=10), sotagliflozin (60 µM; n=10), dapagliflozin (100 µM; n=8), or empagliflozin (100 µM; n=8) for 24 h. **p < 0.01 indicates significant difference from control cells. (One sample *t* test or Wilcoxon signed rank test).

ADAM10 and ADAM17 are proteases that are involved in αKlotho shedding, a process yielding soluble Klotho in the blood or cell culture supernatant (15). To investigate whether ADAM10 or ADAM17 regulation accounts for the different effects of gliflozins on Klotho protein in the cell culture supernatant, we tested the effect of SGLT2 inhibitors on ADAM10 and ADAM17 expression in HK-2 cells. QRT-PCR analyses revealed that none of the SGLT2 inhibitors had a significant effect on *ADAM10* transcripts (**Figure 6A**) in HK-2 cells. However, a significant increase in *ADAM17* transcript levels was observed in response to canagliflozin or sotagliflozin but not dapagliflozin or empagliflozin treatment (**Figure 6B**), an effect likely to contribute to the different actions of SGLT2 inhibitors on Klotho protein in the cell culture supernatant of HK-2 cells.

Finally, we investigated whether the SGLT2 inhibitors also impact on gene expression of *Fgf23*, a bone-derived hormone which requires renal αKlotho as a co-receptor. To this end, UMR-106 osteoblast-like cells were treated without or with SGLT2 inhibitors for 24 h, and *Fgf23* transcripts were determined by qRT-PCR. As a result, control cells exhibited *Fgf23* transcripts levels relative to *Tbp* of 1.0 ± 0.0 arbitrary units (a.u.) (for all: n=3). Treatment with 100 µM dapagliflozin or 100 µM empagliflozin significantly up-regulated *Fgf23* transcript levels (1.8 ± 0.1 a.u.; *p*<0.01 or 2.1 ± 0.2 a.u.; *p*<0.05, resp.), whereas exposure to 60 µM canagliflozin or 60 µM sotagliflozin significantly reduced *Fgf23* gene expression (0.1 ± 0.0 a.u.; *p*<0.01 or 0.4 ± 0.1 a.u.; *p*<0.05, resp.).

## Discussion

Our investigations revealed that common SGLT2 inhibitors used in the treatment of patients with diabetes, CKD, or heart failure modify αKlotho gene expression and Klotho protein secretion in renal MDCK and HK-2 cells.

In canine MDCK cells, canagliflozin, sotagliflozin, and dapagliflozin up-regulated αKlotho gene expression. In contrast, empagliflozin down-regulated αKlotho transcripts in MDCK cells. Importantly, empagliflozin has the highest selectivity for SGLT2 over SGLT1 among the four gliflozins tested (47). According to our quantitative expression analysis, MDCK exhibited low SGLT1 and virtually no SGLT2 expression. Therefore, it can be assumed that exposure to empagliflozin resulted in higher remaining SGLT1 activity compared to exposure to the other SGLT2 inhibitors in MDCK cells. It is tempting to speculate that this difference in remaining SGLT1 activity may play a role in the gliflozin effect on αKlotho in MDCK cells. Certainly, αKlotho up-regulation in MDCK cells was independent of SGLT2 since this transporter was not expressed in MDCK cells. In line with this, cardioprotective effects of SGLT2 inhibitors are not necessarily related to SGLT2, since expression of SGLT2 in the heart and cardiomyocytes is controversial (48–50). Therefore, upregulation of αKlotho expression in MDCK cells by canagliflozin, sotagliflozin, and dapagliflozin is probably also an SGLT2-independent effect.

Therefore, other SGLT2-independent effects of gliflozins must account for their effect on αKlotho in MDCK cells. As a matter of fact, such effects exist and play a role in nephro- and cardioprotection by SGLT2 inhibitors: Among them are anti-inflammatory properties (51, 52). Accordingly, we observed a small, but statistically significant down-regulation of p65 subunit (encoded by *RELA*) of pro-inflammatory NFκB transcription factor (53, 54) by canagliflozin, sotagliflozin, and dapagliflozin. This effect can clearly be expected to be anti-inflammatory and may be one of the SGLT2-independent effects contributing to the up-regulation of αKlotho. Interestingly, in line with its failure to significantly modify αKlotho expression, empagliflozin did not alter *RELA* expression, either. Therefore, suppression of inflammation may be relevant for the up-regulation of αKlotho by SGLT2 inhibitors in MDCK cells in the absence of SGLT2. Alternatively, *RELA* suppression may be secondary to enhancement of αKlotho in canagliflozin-, sotagliflozin-, or dapagliflozin-treated MDCK cells as αKlotho has anti-inflammatory properties (20, 22).

Given the cellular responses of HK-2 cells to SGLT2 inhibitors, the situation even becomes more complex. All SGLT2 inhibitors reduced αKlotho gene expression in HK-2 cells to a small, but statistically significant extent, an observation in contrast to MDCK cells. Our expression analysis suggested markedly higher SGLT2 than SGLT1 expression in HK-2 cells. Since the strong up-regulation of αKlotho in MDCK cells in the absence of SGLT2 expression suggests that this up-regulation is SGLT2-independent, the different expression pattern of SGLT1 and SGLT2 in MDCK cells and HK-2 cells may contribute to the contrasting effects on αKlotho transcripts in the two cell lines. Therefore, inhibition of SGLT2 in HK-2 cells may be accompanied by down-regulation of αKlotho expression.

In the kidney, highest αKlotho expression is observed in the distal tubule, but also the proximal tubule expresses it (55, 56). Whereas MDCK cells are derived from distal tubule, HK-2 cells are of proximal tubule origin (57, 58). The different origin of the two cell lines may also help explain the different cellular response to gliflozin treatment. Since SGLT2 is expressed in proximal tubular cells, the gliflozin effect on SGLT2 and αKlotho may be related to actions in different cell types in the kidney.

Despite the small but significant suppressive action of all four SGLT2 inhibitors on αKlotho transcripts in HK-2 cells, canagliflozin and sotagliflozin enhanced Klotho protein secretion. Hence, both SGLT2 inhibitors ultimately increased Klotho protein levels in the supernatant of HK-2 cells, an effect that can be expected to be in line with up-regulation of αKlotho gene expression by SGLT2 inhibitors in MDCK cells. In contrast, dapagliflozin and empagliflozin did not significantly affect Klotho protein levels in the cell culture supernatant. In the kidney, soluble Klotho is generated due to cleavage of transmembrane αKlotho by ADAM10 and ADAM17 proteinases (15). Our expression analysis of ADAM10 and ADAM17 indeed revealed that canagliflozin and sotagliflozin, but not dapagliflozin or empagliflozin up-regulated ADAM17 expression in HK-2 cells. This effect is likely to explain why canagliflozin and sotagliflozin, but not dapagliflozin or empagliflozin increased Klotho protein concentration in the cell culture supernatant of HK-2 cells although all four gliflozin suppressed αKlotho gene expression in these cells according to qRT-PCR analysis.

Phlorizin, a dual inhibitor SGLT1/SGLT2 not in clinical use since inhibition of both Na^+^-dependent glucose transporters, can be expected to cause serious adverse effects, only had moderate (MDCK cells) or no significant effect (HK-2 cells) on αKlotho although its glucosuric, hence anti-diabetic effect may even be stronger than that of gliflozins. This finding corroborates the notion that the health benefits of SGLT2 inhibitors for the heart or kidneys, which may include up-regulation of αKlotho as unraveled by this study, are independent of the glucose-lowering effects of this class of drugs.

It is a major finding of this investigation that the four SGLT2 inhibitors studied yielded different results in our cell culture experiments: In MDCK cells, empagliflozin failed to up-regulate αKlotho in contrast to canagliflozin, sotagliflozin, and dapagliflozin, and in HK-2 cells, canagliflozin and sotagliflozin, but not dapagliflozin and empagliflozin increased Klotho protein in the cell culture supernatant. It is tempting to speculate whether these findings indeed translate into a different capacity of the SGLT2 inhibitors to upregulate αKlotho levels *in vivo* and whether this would have a clinical significance. Thus far, most clinical studies revealed similar health benefits of different gliflozins. However, it must be kept in mind that the clinical studies rarely include direct comparisons of different gliflozins. In a recent study of acute kidney injury in rats, differences between empagliflozin and sotagliflozin became apparent (59). Whether or not the differences of the four gliflozins with regard to αKlotho matter *in vivo*, must be investigated in the future. A next step could be an *ex vivo* study addressing the effect of the four gliflozins on αKlotho in proximal tubule cells from diabetic rats to investigate whether αKlotho regulation is different in disease.

**Figure 7:**
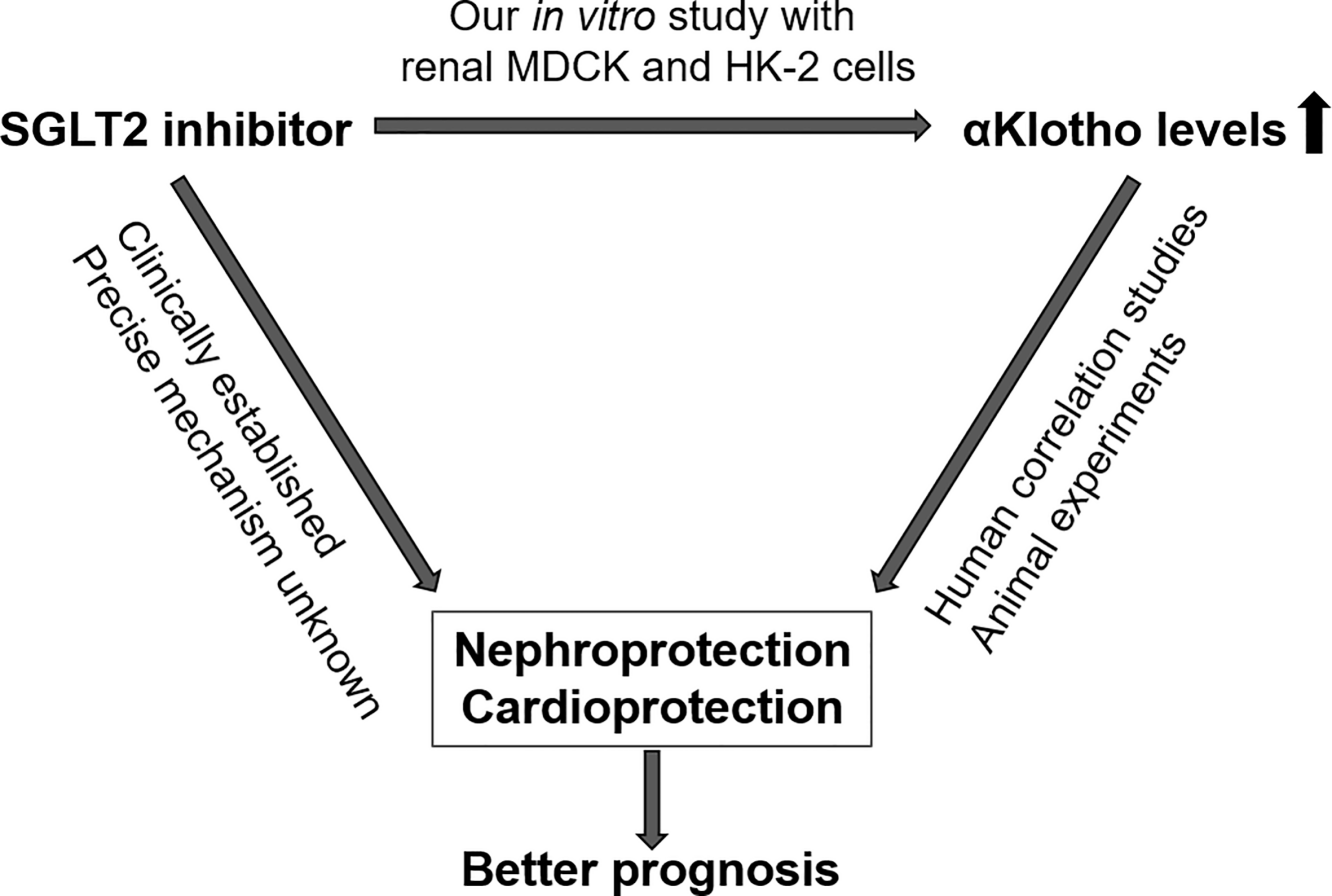
Proposed interdependence of SGLT2 inhibitors and αKlotho with regard to nephro- and cardioprotection.

Our study brings together two novel concepts of nephro- and cardioprotection, i.e. therapy with SGLT2 inhibitors and increasing the availability of αKlotho (**Figure 7**). Given the well-established beneficial effects of gliflozins on the kidney and heart and promising results of Klotho delivery particularly in kidney disease in conjunction with association studies showing better outcome with higher Klotho levels in renal and cardiovascular diseases, our study sheds new light on the relationship between SGLT2 inhibitors and αKlotho levels (46).

Our experiments with UMR-106 cells revealed that the four different SGLT2 inhibitors studied here markedly differed in their effects on FGF23. In humans, treatment with dapagliflozin or canagliflozin elevates serum FGF23 levels, effects at least in part secondary to changes in phosphate metabolism (60, 61). Why canagliflozin and sotagliflozin directly decreased and dapagliflozin and empagliflozin directly increased *Fgf23* gene expression in cell culture experiments remains enigmatic and must be clarified in future studies.

It is a major limitation of our study that it is solely cell culture-based. Clearly, additional animal experiments and human studies are warranted to decipher the effect of SGLT2 inhibitors on αKlotho *in vivo*. Furthermore it would be interesting to study whether other antidiabetics also impact on αKlotho.

Taken together our study revealed distinct actions of four different SGLT2 inhibitors on αKlotho and ADAM17 gene expression and Klotho protein secretion in two different renal cell lines, distal tubular MDCK cells and proximal tubular HK-2 cells.

## Data availability statement

The raw data supporting the conclusions of this article will be made available by the authors, without undue reservation.

## Author contributions

MFe and MFö designed the research. MFe, MFö, and LW interpreted data. MFe and MFö wrote the manuscript. MFe and LW performed the research and analyses. All authors contributed to the article and approved the submitted version.
